# Electronic State Unfolding for Plane Waves: Energy Bands, Fermi Surfaces, and Spectral Functions

**DOI:** 10.1021/acs.jpcc.1c02318

**Published:** 2021-06-09

**Authors:** David Dirnberger, Georg Kresse, Cesare Franchini, Michele Reticcioli

**Affiliations:** † Faculty of Physics and Center for Computational Materials Science, University of Vienna, 1090 Vienna, Austria; ‡ VASP Software GmbH, 1090 Vienna, Austria; § Dipartimento di Fisica e Astronomia, Università di Bologna, 40127 Bologna, Italy

## Abstract

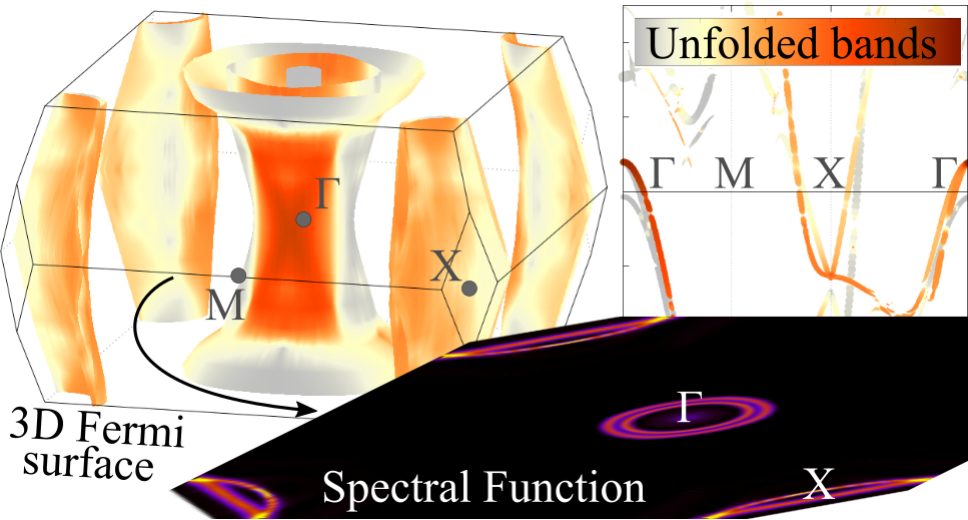

Present day computing facilities allow for first-principles density functional theory studies of complex physical and chemical phenomena. Often such calculations are linked to large supercells to adequately model the desired property. However, supercells are associated with small Brillouin zones in the reciprocal space, leading to folded electronic eigenstates that make the analysis and interpretation extremely challenging. Various techniques have been proposed and developed to reconstruct the electronic band structures of super cells unfolded into the reciprocal space of an ideal primitive cell. Here we propose an unfolding scheme embedded directly in the Vienna *Ab initio* Simulation Package (VASP) that requires modest computational resources and allows for an automatized mapping from the reciprocal space of the supercell to the primitive cell Brillouin zone. This algorithm can compute band structures, Fermi surfaces, and spectral functions by using an integrated postprocessing tool (bands4vasp). Here the method is applied to a selected variety of complex physical situations: the effect of doping on the band dispersion in the BaFe_2(1–*x*)_Ru_2*x*_As_2_ superconductor, the interaction between adsorbates and polaronic states on the TiO_2_(110) surface, and the band splitting induced by noncollinear spin fluctuations in EuCd_2_As_2_.

## 1 Introduction

Material science simulations adopting periodic boundary conditions in the framework of the density functional theory (DFT) may require large unit cells to model long or broken periodicity in crystals. Supercells (i.e., large unit cells built by the stacking of smaller primitive cells forming an ideal Bravais lattice) are used to study the effects of lattice impurities (e.g., local dislocations, defects, doping) and also to investigate domain boundaries, magnetic orders, surface reactivity, and structural reconstructions, just to name a few common applications.^1,2^ Whereas well-developed facilities and efficient DFT packages are capable of dealing with hundreds and even thousands of atoms in large cells, the analysis of the electronic properties (such as the energy band structure or the Fermi surface) gets complicated by the shrinking of the Brillouin zone (BZ) and the consequent folding of the eigenstates in the reciprocal space.^3^ This also prevents a genuine comparison with photoemission spectroscopy experiments.^4,5^

**Figure 1:**
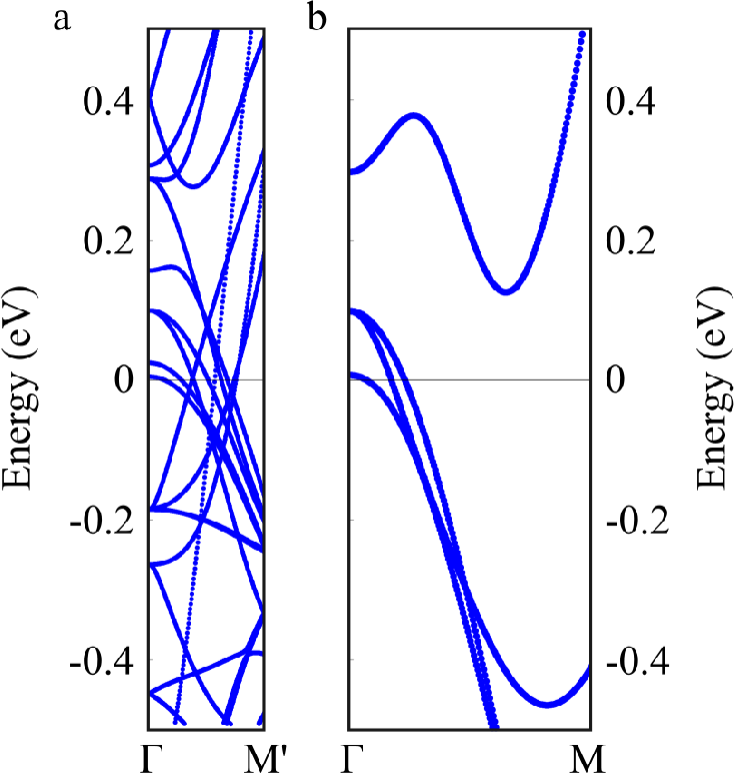
Example of eigenstate folding for the pristine BaFe_2_As_2_ compound. Band structures obtained by using (a) a supercell and (b) a primitive cell.

Figure 1 shows an example of the intricate band structure typically obtained by using a supercell: Clearly, the bands calculated by using the primitive cell instead allow for a more straightforward analysis. The intricate supercell states can be unfolded back into the larger BZ of the primitive cell by applying the unfolding technique.^3,6−10^ This technique is based on the projection *P*_***K****m*_ of the supercell eigenstates |***K****m*⟩ on the primitive cell eigenstates |***k****n*⟩
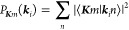
1where *m* and *n* denote energy band indices at vectors ***K*** and ***k***_*i*_ in the reciprocal space of the supercell and the primitive cell, respectively. This projection represents the amount of Bloch character of the states |***k***_*i*_*n*⟩ contributing to |***K****m*⟩, which allows for a direct connection between the reciprocal space of the supercell and the primitive cell: This assignment is straightforward for supercells built as a perfect stacking of the primitive cell because the Bloch character is zero for all but one |***k***_*i*_*n*⟩ state; conversely, nontrivial supercells modeling deviations from the primitive cell symmetry (induced by, for example, impurities and disorder) show faded Bloch characters, with multiple contributions from the primitive cell states to the |***K****m*⟩ state. By weighting the contributions of all single states with *P*_***K****m*_, it is indeed possible to obtain an effective band structure (EBS) of the supercell unfolded in the larger BZ of the primitive cell.

If the eigenstates |***K****m*⟩ are expanded in terms of a plane-wave basis set with coefficients *C*_*m*,***K***_, then eq 1 can be rewritten in terms of states of the supercell only^11^
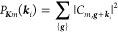
2By expressing *P*_***K****m*_ in this form, all information required from the primitive cell is purely geometric and collected by the reciprocal lattice vector ***g*** applied to the ***k***_*i*_ vectors in the reciprocal space. This alternative formulation has the advantage of avoiding any calculation on the primitive cell as well as any direct comparison between the two spaces, which could turn out to be technically challenging.

The Vienna *Ab initio* Simulation Package (VASP) is an optimal candidate for the implementation of unfolding calculations. This code can efficiently deal with large cells, and plane waves are used as basis functions. Moreover, a basic implementation of eq 2 is already available in the recent VASP releases.^11^ In this work, we extend the original unfolding scheme aiming to reduce the memory requirements and to simplify the user interface for both the input parameters and the extraction of output data. Specifically, we implemented an automatized scheme for generating the supercell reciprocal-space vectors ***K*** starting from the given ***k***_*i*_ vectors in the primitive cell space. The calculation of the *P*_***K****m*_ projection can be limited only to the automatically determined (***K***, ***k***_*i*_) pairs of interest, saving memory resources in the calculation. The user interface has been simplified, including an automatic initialization of the primitive cell; moreover, the user is provided with a postprocessing package for the analysis of the results, “bands4vasp”,^12^ which implements the possibility of graphically visualizing the unfolded energy band structure and spectral functions and includes an algorithm that can determine Fermi wave vectors and Fermi surfaces from folded states with faded Bloch character.

This updated implementation of the unfolding technique in VASP can be applied to a wide range of physical problems: In the following, we describe examples of such applications, starting with an analysis of the electronic states of the Ru-doped BaFe_2_As_2_ superconductor, a benchmark test to show the reliability and main features of our packages. We continue with an application focused on a novel aspect in the field of catalysis and surface reactivity, the interplay of surface adsorbates with small polarons (i.e., strongly localized in-gap states coupled to lattice phonons). Here the interaction between CO molecules and polarons on the TiO_2_(110) surface is revealed by the unfolding scheme, which resolves the small perturbations induced by the adsorbates on the otherwise perfectly flat polaronic bands. Finally, we address the effects of spin fluctuations on the electronic states of EuCd_2_As_2_: By modeling the phase transition from a noncollinear paramagnetic ordering to ferromagnetic domains and to a complete ferromagnetic order, the progressive removal of the energy degeneracy of the states close to the Fermi level is revealed by means of spectral functions obtained in the framework of the unfolding.

## 2 Methodology

**Figure 2:**
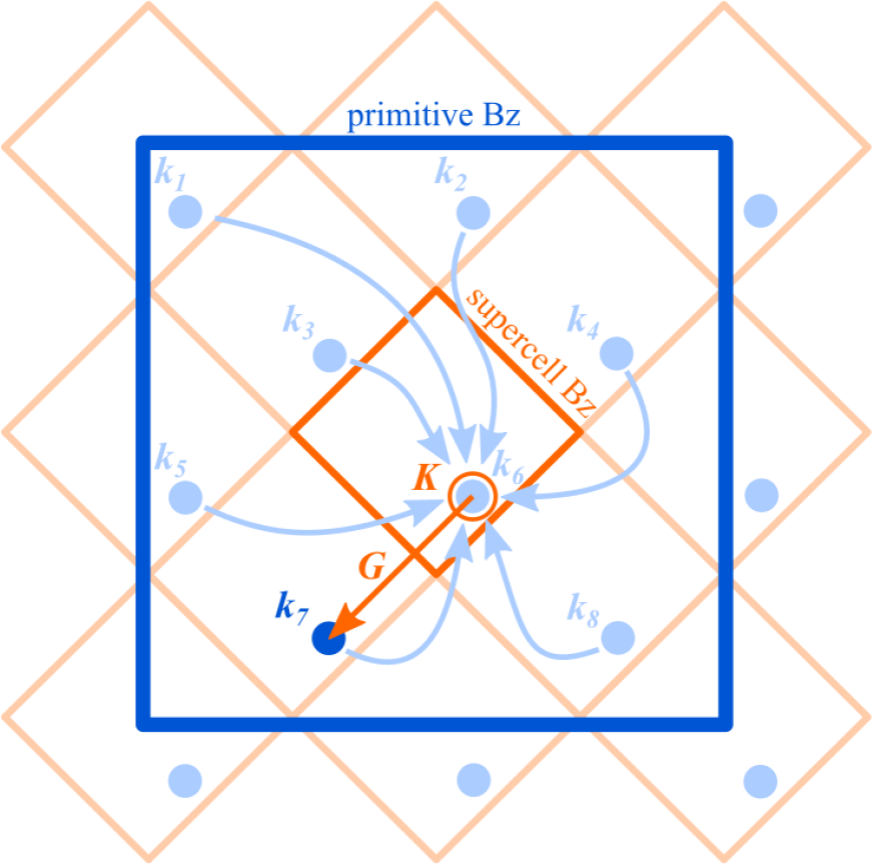
Sketch of the folding problem for a bidimensional 2√2 × 2√2 supercell. Primitive cell BZ and supercell BZ are represented by large blue and small orange squares, respectively. The arrows indicate the folding of eigenstates from the |***M***_2√2×2√2_| = 8 different ***k***_*i*_ primitive cell vectors (filled circles) into one ***K*** supercell vector (open, orange circle). The reciprocal lattice vector ***G*** highlights the eigenstates unfolding from the ***K*** supercell vector to a selected ***k***_*i*_ primitive cell vector.

Electronic eigenstates calculated for supercells can be unfolded into the reciprocal space of the primitive cell by using external packages based on different methods^13−17^ or directly in VASP, which implements eq 2.^11,18−20^ We have optimized the original implementation in VASP by carefully considering the relation between the ***K*** and ***k***_*i*_ vectors, as discussed as follows and sketched in Figure 2. At the end of this section, we report a brief description of the bands4vasp postprocessing package^12^ that includes convenient visualization tools as well as an algorithm for the automatic identification of Fermi surfaces from supercell folded states with faded Bloch characters.

The supercell and the primitive cell are described in terms of 3 × 3 matrices (***A*** and ***a***, respectively) constructed by the corresponding lattice vectors; similarly, the BZs are defined by matrices (***B*** and ***b***) built by the reciprocal lattice vectors. A transformation matrix ***M*** with integer elements relates the supercell and primitive cell in both the direct and reciprocal spaces^19^
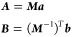
3The determinant |***M***| of the transformation matrix defines the ratio between the supercell and the primitive cell volumes in the direct (*V* and *v*) and reciprocal (*W* and *w*) spaces: |***M***| = *V*/*v* = *w*/*W*. The eigenstates of a single ***K*** point in the supercell BZ correspond to eigenstates of the primitive cell folded from different primitive cell points ***k***_*i*_, with *i* running from 1 to |***M***|. (See Figure 2). These points are connected by linear combinations of supercell reciprocal lattice vectors {***G***}_*i*_
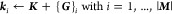
4The folding problem can be equivalently expressed by considering that the eigenstates of one ***k***_*i*_ vector fold to one unique ***K*** point in the first BZ of the supercell, determined by one specific combination of supercell reciprocal lattice vectors {***G***}_0_^7^
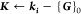
5See also the straight arrow in Figure 2.

The two previous equations allow for an efficient mapping of the supercell and primitive cell reciprocal spaces. We implemented the possibility of limiting the calculation of the Bloch character to specific (***K***, ***k***_*i*_) pairs fulfilling eq 4 for any supercell ***K*** vector defined in the input, excluding all other pairs that would trivially result in *P*_***K****m*_(***k***_*i*_) = 0. This restriction considerably reduces the computational effort, as fewer Bloch characters need to be evaluated: The number of evaluated characters for states on any ***K*** is given by |***M***|.

Additionally, the calculation of the Bloch character can be further limited to selected ***k***_*i*_ vectors of interest. In fact, the user is typically interested in retrieving the eigenstates for selected ***k***_*i*_ vectors from the folded supercell states rather than exploring all contributions to the supercell ***K*** vectors. Therefore, the ***k***_*i*_ vectors can be initialized by the user; then, they are automatically translated into the supercell reciprocal space by the transformation
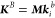
6where ***K***^*B*^ and ***k***_*i*_^*b*^ represent the vector coordinates expressed in the supercell and primitive cell reciprocal spaces, respectively. The calculation of the Bloch character can then be executed as in the original implementation, but it is limited to (***K***, ***k***_*i*_) pairs satisfying eq 5. This approach drastically reduces the computational effort of the algorithm because the *P*_***K****m*_(***k***_*i*_) character needs to be evaluated on only one single ***K*** for any given ***k***_*i*_. (See also the discussion in the Benchmark and Results section.)

The implementation of these automatized features simplifies the initialization of the unfolding calculation for the user. Moreover, the primitive cell lattice vectors can also be automatically determined from the supercell by the program, by simply inverting eq 3, if the transformation matrix ***M*** is specified in input: ***a*** = ***M***^–1^***A***.

The extraction of the output data is quite straightforward as well. To further facilitate the analysis of unfolding calculations, we make available the bands4vasp postprocessing package for band structure analysis.^12^ This can also be used for the construction of unfolded band structures, Fermi surfaces, and spectral functions, as well as the automatic calculation of Fermi wave vectors (i.e., the ***k***_*i*_ vector of eigenstates at the Fermi level). We recall that in the framework of unfolding calculations, the spectral function *A* is approximated as
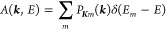
7where δ(*E*_*m*_ – *E*) are Dirac delta functions centered around *E*_*m*_ energies.

The calculation of Fermi wave vectors needs special consideration in unfolding calculations for supercells that are not built as a perfect stacking of the primitive cell. In this nontrivial case, multiple (up to |***M***|) primitive cell states |***k***_*i*_*n*⟩ contribute to the supercell state |***K****m*⟩, as revealed by faded *P*_***K****m*_(***k***_*i*_) Bloch characters, typically leading to broad unfolded bands. To identify the crossing of these broad bands with the Fermi level, we adopt a simple procedure: (i) For every ***k***_*i*_ vector, the energy eigenvalues in an arbitrary energy range are assigned to a certain energy band by also considering similarities in the orbital symmetry of the eigenstates. (ii) Then, we define a band center of mass by averaging the energy values with weights given by the corresponding *P*_***K****m*_(***k***_*i*_) character. (iii) Finally, the intersection of every band with the Fermi level is found by interpolation. An example of the automatic calculation of Fermi wave vectors and the corresponding Fermi surface is discussed in the next section.

## 3 Benchmark and Results

We tested our implementation of the unfolding algorithm embedded in VASP by considering the electronic properties of BaFe_2(1–*x*)_Ru_2*x*_As_2_ (with *x* = 0 and 0.25 for the undoped and doped cases, respectively). This material is indeed a good testbed because much reference data are available in literature.^19−21^ We also take this opportunity to describe features included in the postprocessing bands4vasp package, such as the visualization of band structures, the Fermi surfaces and spectral function, and the calculation of Fermi wave vectors (Section 3.1). Finally, we show novel and more challenging applications of our approach: By performing calculations on large cells modeling the TiO_2_(110) surface, the unfolding analysis reveals the formation of flat bands originating from in-gap polaronic states that are perturbed by the interaction with CO adsorbates deposited on the material surface (Section 3.2). Moreover, we describe the band splitting occurring in the transition process from noncollinear paramagnetic to ferromagnetic ordering in EuCd_2_As_2_ (Section 3.3).

### 3.1 Analysis of Metallic States in BaFe_2(1–*x*)_Ru_2*x*_As_2_

The metal-to-superconductor transition driven by Ru doping in the BaFe_2_As_2_ pnictide has attracted wide interest in both theoretical and experimental communities,^22−29^ and the unfolding technique has proven itself useful to support DFT investigations. One of the most evident results observed from the EBS is the progressive closure of hole pockets upon doping due to a coupling with structural distortions.^20,21^

We performed spin-unpolarized DFT calculations on BaFe_2_As_2_ by maintaining a similar computational setup as in ref (19) but by applying the updated version of the unfolding algorithm. We modeled the BaFe_2_As_2_ structure adopting supercells of different size (***A***_2_, ***A***_8_, ***A***_16_), constructed by applying the following transformation matrices (as described in eq 3)
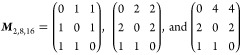
with determinants |***M***| = 2, 8, 16, respectively. The ***M***_2_ matrix represents the transformation from the primitive cell to the conventional unit cell for compounds with the body-centered tetragonal *I*4/*mmm* space group, such as BaFe_2_As_2_.^30^ We remark that the ***M*** matrix elements are required to be integers in the unfolding formalism, but this requirement does not prevent us from modeling rotations or nontrivial transformations.^9^ In general, no additional theoretical constraint is introduced by our implementation.

**Figure 3:**
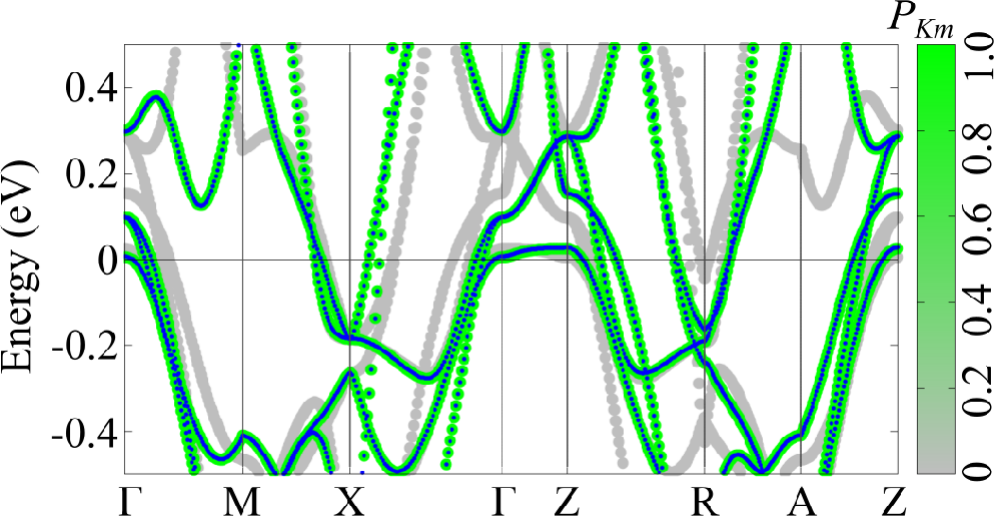
Effective band structure of the supercell constructed by the ***M***_2_ transformation matrix (gray-to-green color gradient) compared with the band structure of the primitive cell (blue).

We performed a preliminary test by considering the pure (undoped) BaFe_2_As_2_ crystal and comparing the EBS obtained from the supercell with the bands calculated directly for the primitive cell. In fact, no difference should be observed between the two band structures once the supercell states are unfolded into the reciprocal space of the primitive cell. Figure 3 shows the unfolded bands of the supercell ***A***_2_ constructed by the transformation matrix ***M***_2_. As expected (due to |***M***_2_| = 2), we obtained for the supercell twice as many bands as obtained directly from the primitive cell calculation, shown in the figure for comparison. The unfolding algorithm is able to correctly identify the band folding and to assign the Bloch character *P*_***K****m*_ accordingly: The gray color in the gradient palette identifies the folded bands with *P*_***K****m*_ = 0 that belong to different points in the primitive cell reciprocal space. Usually, the folded bands should not be considered in the EBS, and can be omitted from the graph (e.g., by setting the gradient palette with *P*_***K****m*_ = 0 to the same color as the background of the image).

**Figure 4:**
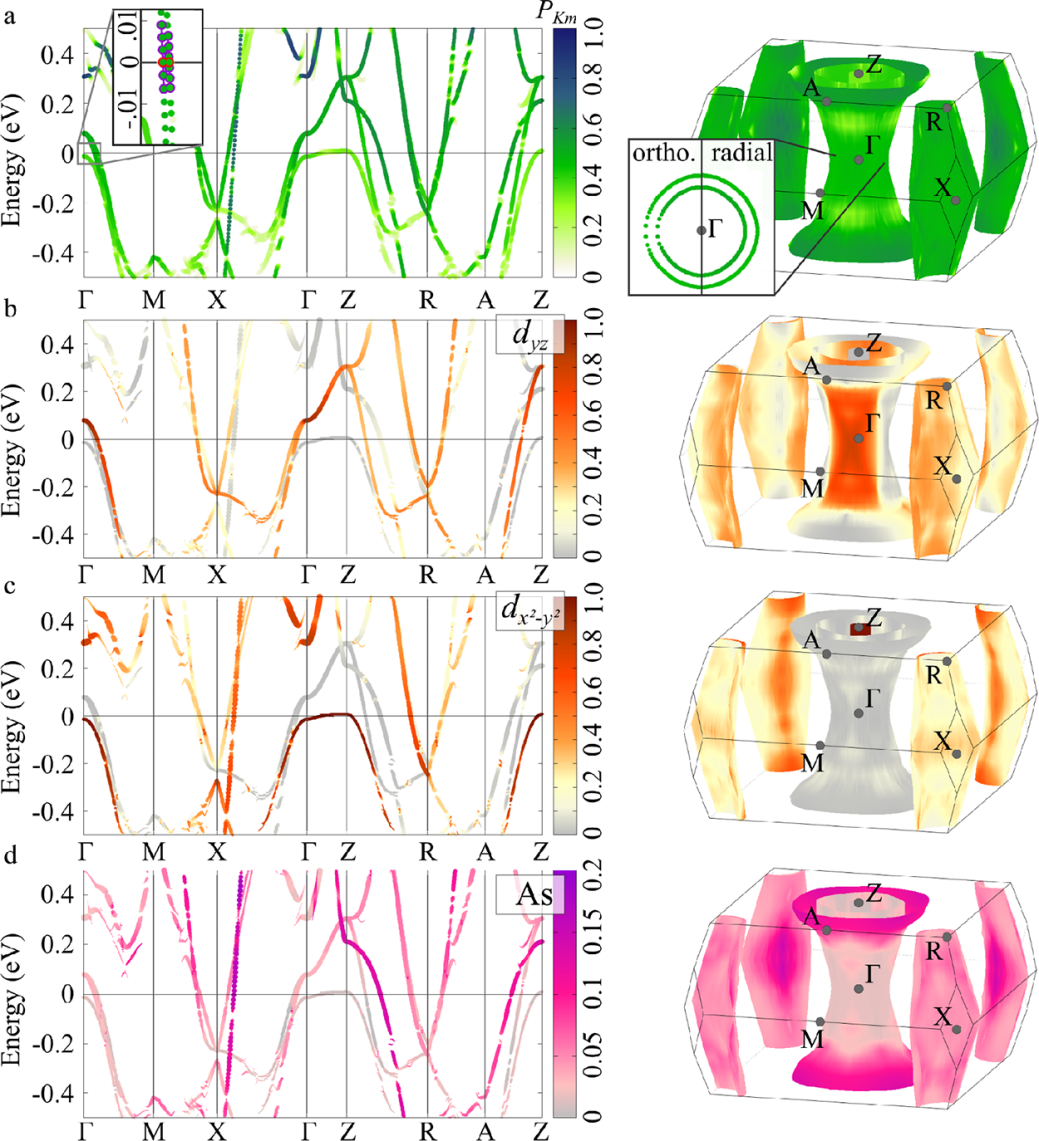
Unfolding for the doped BaFe_2(1–*x*)_Ru_2*x*_As_2_ (*x* = 0.25). (a) Effective band structure and 3D Fermi surface. The color gradient indicates the Bloch character. The insets show the identification of the Fermi wave vectors and a 2D cut of the Fermi surface with orthogonal (left) and radial (right) sampling of the reciprocal space. (b–d) Band structures with the Bloch character represented by a variable point sizes with the color gradient indicating the (b) d_*xz*_ and (c) d_*x*^2^–*y*^2^_ orbital character of Fe atoms and (d) the overall contribution from As atoms. The corresponding 3D Fermi surfaces are also shown.

Figure 4 shows the effects of 25% of Ru doping on the electronic properties of BaFe_2(1–*x*)_Ru_2*x*_As_2_ (*x* = 0.25). The band structure in Figure 4a correctly reproduces the closure of the hole pockets around the Γ and *Z* high-symmetry points.^20^ The intersection of the bands with the Fermi level can be automatically identified by using the bands4vasp tool: The inset in Figure 4a shows the assignment of the eigenstates around the Fermi energy to different bands (by considering the Bloch character and the orbital symmetry) and the intersection of every band with the Fermi level found by interpolation. This feature is extremely useful for the analysis of the Fermi wave vectors, which get progressively shortened for the hole pockets around Γ upon Ru doping in this material. Moreover, by collecting all Fermi wave vectors in the BZ, it is possible to construct 3D Fermi surfaces. (See the right panel in Figure 4a.) The 2D cut of the basal plane around Γ (see the inset in Figure 4a) highlights the importance of an accurate sampling of the reciprocal space by comparing the Fermi surface obtained using two different approaches. The right side of the Fermi surface is constructed by using a radial distribution of the *k* points, leading to a better resolved description of the states around Γ as compared with the resolution obtained by adopting a conventional rectangular grid (left side).

The bands4vasp tool can also extract the atomic orbital character of the eigenstates from unfolding calculations. Figure 4b–d shows the EBSs with Bloch character represented by the size of the circles and the color gradient representing the projection of the states on the d_*yz*_ (Figure 4b) and d_*x*^2^–*y*^2^_ (Figure 4c) orbitals (essentially due to Fe atoms) and the overall contribution from As atoms (Figure 4d). The contribution of Fe states around Γ (d_*yz*_ and d_*xz*_ (not shown here)) and *Z* (d_*x*^2^–*y*^2^_) and on the BZ border clearly stems from both the band structure and the corresponding 3D Fermi surfaces. Similarly, the hybridization with the As atoms leads to states at the Fermi level around *Z* and *X*.

**Figure 5:**
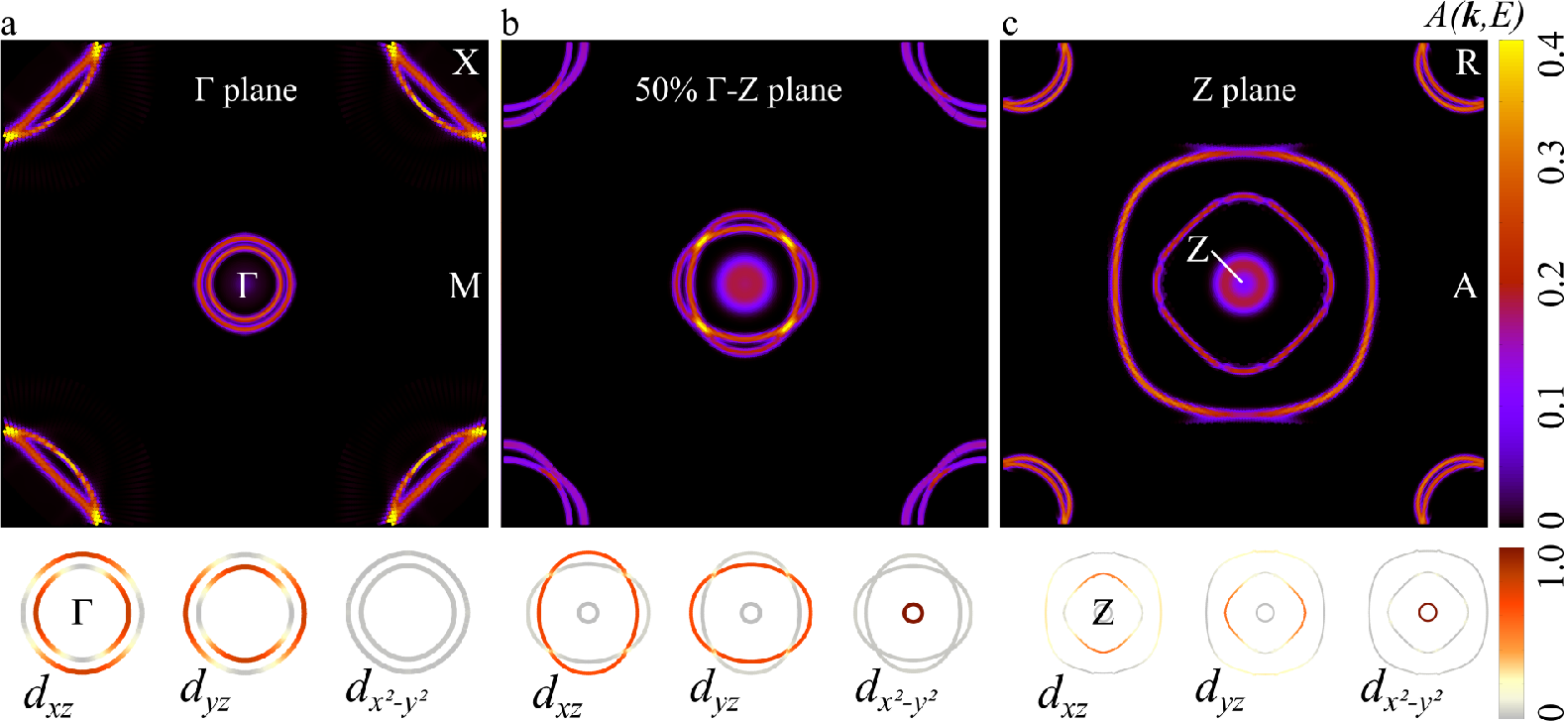
2D Fermi surfaces calculated using the spectral function *A*(***k***, *E*_Fermi_) for (a) the basal plane and (b) the parallel planes halfway in the Γ–*Z* direction and including (c) the high-symmetry point *Z*. The corresponding orbital characters of the central rings are shown in the bottom of each panel.

By looking carefully at the d_*x*^2^–*y*^2^_ orbital (in Figure 4c), we note a band crossing the Fermi level in the Γ–*Z* direction. This feature is highlighted in Figure 5, which shows 2D Fermi surfaces for the basal plane and the parallel planes along Γ–*Z* obtained for the spectral function *A*(***k***, *E*_Fermi_). On the basal plane, only two states appear sharply around Γ (d_*xz*_ and d_*yz*_; see the orbital analysis on the bottom images in Figure 5a). Conversely, the spectral function for the inner state (with d_*x*^2^–*y*^2^_ orbital symmetry) is absent and becomes progressively better defined when moving toward *Z* (Figure 5b,c). The spectral function also allows us to easily identify band degeneracy and crossing points between different bands, which are revealed by a more intense value. In Figure 5b, the crossing of the d_*xz*_ and d_*yz*_ states determines four points with a large value for the spectral function around the center of the plane. This crossing corresponds to a progressive band switching between d_*xz*_ and d_*yz*_ states, clearly identifiable by looking at the evolution of the orbital symmetry of the internal and external rings moving from Γ to *Z*.

We conclude our benchmark by commenting on the memory requirements of the unfolding algorithm. The automatic determination of (***K***, ***k***_*i*_) pairs (as in eqs 5 and 6) reduces the computational effort by limiting the unfolding procedure only to points of interest. For small systems, such as the ***A***_2_ supercell, we counted a memory gain of ∼20% when using 300 ***k*** points, which increased to approximately 30 and 50% for the larger ***A***_8_ and ***A***_16_ cells, respectively. The lower computational requirements are very useful for performing unfolding calculations that model complex systems. Some original applications of the unfolding method are presented in the following sections.

### 3.2 In-Gap Polaronic States and Adsorbates on the TiO_2_(110) Surface

**Figure 6:**
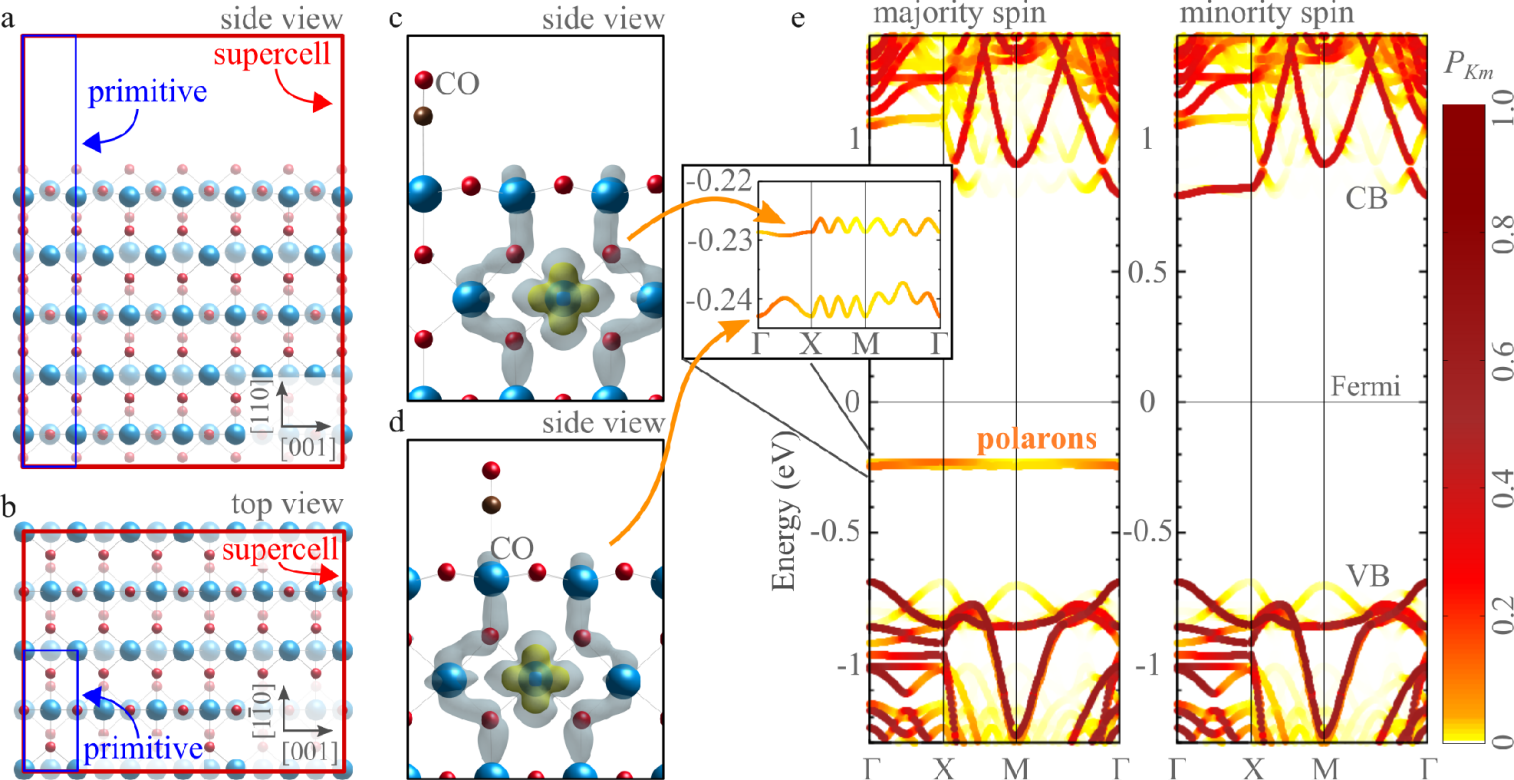
Unfolding for surface slab calculations. (a) Side and (b) top views of the pristine rutile TiO_2_(110) surface. The blue and red rectangles indicate the primitive cell and the 6 × 2 super cell, respectively. (c,d) Details of the two polarons and the two adsorbed CO molecules. The gray and yellow areas represent the polaronic charge at different isosurface levels. (e) Corresponding effective band structure. The inset shows the detail of the polaronic bands between the conduction (CB) and valence (VB) bands.

The unfolding algorithm can also be applied for DFT calculations on systems with reduced dimensionality, such as the surface slab shown in Figure 6, modeling the pristine rutile TiO_2_(110) termination. Unit cells modeling surfaces of solids in VASP contain a vacuum region to interrupt the periodicity of the system along the surface normal.^31^ Typically, several atomic layers are also included in the model to mimic the properties of the bulk below the surface. (See Figure 6a.) Primitive cells and supercells share the same vector perpendicular to the surface, as in Figure 6a. For the modeling of surface reconstructions or defects, supercells are constructed by enlarging the lateral size (see Figure 6b), leading to folding of electronic states by analogy to the bulk.

Rutile TiO_2_(110) supercells can be used to model the formation of oxygen vacancies on the surface that lead to the stabilization of small electron polarons, that is, electrons strongly localized on Ti ions and coupled to the phonon field.^32−34^ Small polarons are typically associated with eigenstates appearing in the energy band gap of semiconductors; moreover, polaron localization can occur on different sites with different formation energies.^35^ (In rutile, subsurface Ti ions are preferred over surface sites.) Polarons are known to drastically affect the electronic and chemical properties of the hosting material, with a substantial impact on the applications. We focus here on the chemical activity of the rutile surface by considering the interplay between polarons and CO adsorbates, recently proposed as the key mechanism for the CO adsorption process on this material.^36^ The analysis of the unfolded band structure discussed as follows reveals perturbations of the polaronic states that appear as a fingerprint of the polaron–adsorbate interaction.

Figure 6c–e collects the results obtained for large 6 × 2 supercells containing 363 atoms, including two CO molecules and two polarons. (Technical details of the calculation can be found in ref (36).) As shown in Figure 6c,d, the CO can adsorb on Ti sites at different distances from the polarons. The corresponding EBS (unfolded on the surface primitive cell) shows the appearance of the strongly localized polaronic states, revealed by two flat in-gap bands (one per polaron) in the majority spin channel (Figure 6e). By looking closer at these in-gap bands (inset in Figure 6e), we note that the two polaronic states are not degenerate due to the interaction with the CO molecules. The band appears more perturbed for the polaronic state closer to the CO molecule, as manifested by the increased bandwidth. It is expected that perturbations of the polaronic in-gap states may also originate from the repulsive interaction of polarons at a small distance. In general, a detailed analysis of polaronic and strongly localized electronic states via eigenstate unfolding in supercell calculations might facilitate the interpretation of in-gap states in spectroscopy measurements.^34,37,38^

### 3.3 Noncollinear Ferromagnetic Fluctuations in EuCd_2_As_2_

We describe here the application of the unfolding algorithm on systems with noncollinear magnetic ordering.^15^ We consider the paramagnetic-to-ferromagnetic transition in EuCd_2_As_2_, an interesting semimetal showing the emergence of Weyl fermions in the paramagnetic phase due to spin fluctuations of Eu magnetic moments.^39^ Here we focus on the analysis of the electronic properties of the states around Fermi by considering different magnetic orders and by calculating the corresponding spectral functions, which allows for a clear description of the energy band degeneracy.

**Figure 7:**
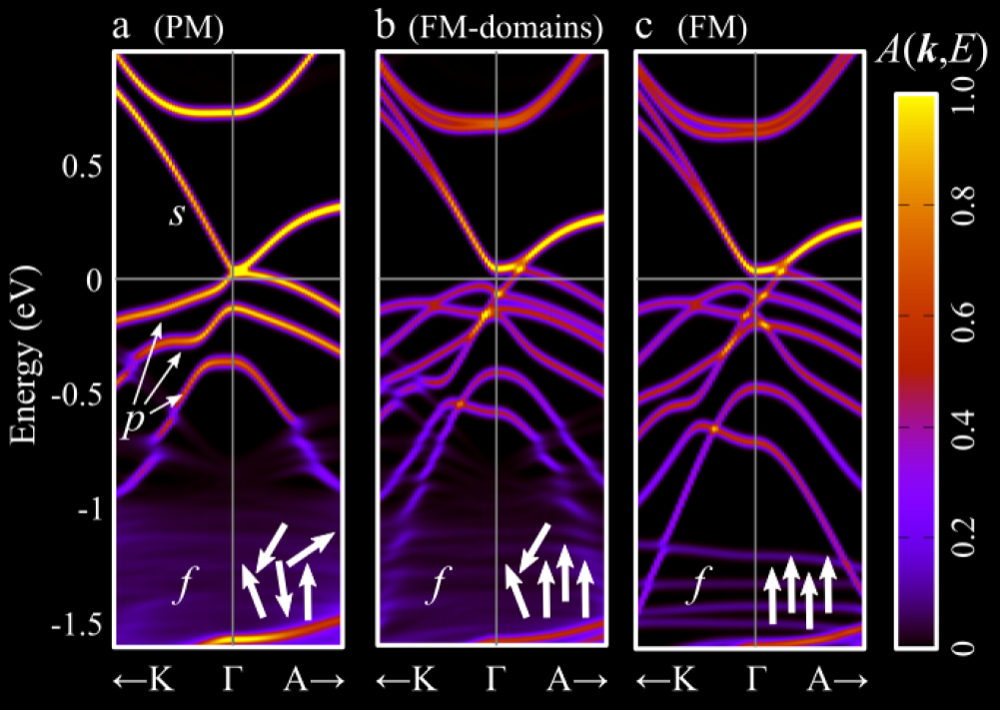
Effective band structure obtained by noncollinear-spin calculations for EuCd_2_As_2_ considering (a) a paramagnetic phase, (b) ferromagnetic domains, and (c) perfect ferromagnetic ordering. The band structures focus around the Γ point from the *K*–Γ–*A* path. The white arrows sketch the arrangement of the magnetic moments of the Eu atoms.

Figure 7 compares the effective spectral functions calculated for EuCd_2_As_2_ with different magnetic orderings. (Technical details of the calculations are described in ref (39).) The paramagnetic phase was modeled by a large supercell including 16 Eu atoms with magnetic moments fixed to random orientations, resulting in a vanishing total magnetization: The corresponding spectral function unfolded in the reciprocal space of the primitive cell is shown in Figure 7a. The flat f bands of Eu atoms appearing around −1.5 eV show an evident incoherence due to the random orientation of the magnetic moments. The three p bands related to the As atoms appear strongly spin-degenerate: The spectral function is very effective in capturing the band degeneracy, as degenerate bands result in higher values of the spectral character, integrating the contribution from every state, as described in eq 7 (at variance with band structures, instead showing the Bloch character of every state individually).

The ferromagnetic phase shows interesting changes (Figure 7c). First, we note that the f bands are more coherent, as expected, due to the ferromagnetic alignment of all Eu magnetic moments. Remarkably, the ferromagnetic order induces a splitting of the p states of As atoms: We indeed observe six bands, lifting the spin degeneracy of the three p bands in the paramagnetic phase. (Note also the lower spectral function value as compared with the paramagnetic case.)

Although the study of ferromagnetic systems could be done directly in the primitive cell, the supercell approach allowed us to study the paramagnetic-to-ferromagnetic transition by considering ferromagnetic domains embedded in a paramagnetic environment. In Figure 7b, we show the spectral function of the system including a large ferromagnetic domain (consisting of 10 Eu atoms with aligned magnetic moments) and a smaller region (6 atoms) with Eu magnetic moments constrained to random directions. The splitting of the p orbitals persists in this transition state: This is an example of the effect of spin fluctuations on the paramagnetic phase of the compounds. In smaller ferromagnetic domains, the band splitting is gradually reduced, progressively converging toward the paramagnetic degeneracy. (Results obtained by modeling different sizes of the ferromagnetic domain are available in ref (39).)

## 4 Conclusions

In summary, we report here our optimization of the unfolding scheme embedded in VASP, characterized by a simplified user interface and reduced memory requirements, thanks to an efficient mapping between the reciprocal spaces of the supercell and primitive cells. The construction of EBSs, spectral functions, Fermi surfaces, and projections of electronic states on orbitals and ions is further facilitated by the bands4vasp postprocessing package.

The unfolding scheme is extremely useful in the interpretation of the results obtained by supercell approaches, and it facilitates the comparison with the experimental observations, especially in the field of spectroscopy. The application range is very broad. We take here the BaFe_2(1–*x*)_Ru_2*x*_As_2_ superconductor as a benchmark, given the large amount of data available in the literature. Moreover, we considered the adsorption of CO molecules on the rutile TiO_2_(110) surface to show the suitability of the algorithm for very large supercells, such as those required in surface science calculations. In this case study, the EBS highlights the interactions of adsorbates with strongly localized polarons, revealed by the perturbation of the flat polaronic bands. Finally, we performed noncollinear calculations for the EuCd_2_As_2_ semimetal. The supercell approach allowed us to model the paramagnetic phase by constraining magnetic moments along random directions, resulting in a vanishing total magnetization. The corresponding spectral function reveals the spin degeneracy of shallow states below the Fermi level that is lifted by including spin fluctuations via the formation of ferromagnetic domains.

The implementation of the unfolding algorithm proposed here represents a useful computational tool for a wide range of physical and chemical phenomena requiring very large supercells. Thanks to the improved interface, the reduced computational requirements, and the integrated analysis package, these now become easily accessible.
